# Quantitative Loop-Mediated Isothermal Amplification (qLAMP) for the Rapid Discrimination of Normal and Cancerous Tissue Models: An Arduino-Based Portable Cancer Detection System Assisted by a pH Microelectrode

**DOI:** 10.3390/bios16080453

**Published:** 2026-08-20

**Authors:** Sergio Bravo-González, Luisa María Reyes-Cortés, Kristen Aideé Pérez-Alvarez, Grissel Trujillo-de Santiago, Mario Moisés Álvarez

**Affiliations:** 1 Centro de Biotecnología-FEMSA, Escuela de Ingeniería y Ciencias, Tecnológico de Monterrey, Ave. Eugenio Garza Sada 2501, Monterrey 64700, NL, Mexico; a01191795@exatec.tec.mx (S.B.-G.); a01032401@tec.mx (L.M.R.-C.); a00829551@exatec.tec.mx (K.A.P.-A.); 2 Departamento de Mecatrónica, Escuela de Ingeniería y Ciencias, Tecnológico de Monterrey, Ave. Eugenio Garza Sada 2501, Monterrey 64700, NL, Mexico; 3 Advanced Biofabrication, Expedition-FEMSA, Tecnológico de Monterrey, Ave. Eugenio Garza Sada 2501, Monterrey 64700, NL, Mexico

**Keywords:** LAMP, point-of-care (POC), biomarker, cancer, diagnostic, spheroid

## Abstract

Cancer, the second leading cause of death worldwide, is a significant global challenge, and widespread, accessible, and early diagnostics are recognized as the most cost-effective strategies for reducing cancer burdens. Point-of-care (POC) systems offer an attractive alternative by enabling rapid and cost-effective diagnoses. We introduce a novel POC strategy for cancer biomarker identification based on monitoring the isothermal amplification of relevant cancer markers using a portable Arduino-based loop-mediated isothermal amplification (LAMP) system. The trajectory of the LAMP reaction during the first 3 min of the reaction is used as an indicator of the rate of amplification (defined as the m_P3_ value). We obtained sets of m_P3_ values that showed statistically significant differences in the genetic expression of four genes (*ESR 1*, *PGR*, *Her2*, and *Ki67*) within and between tissue spheroids derived from the MCF7, MDA-MB-231, Du145, and BJ fibroblast cell lines. We then used principal component analysis and clustering techniques to demonstrate that the m_P3_ value sets derived from the expression of the four selected genes are sufficient to distinguish tissue spheroids derived from four different commercial cell lines. Further qPCR and immunostaining assays confirmed the quantitative LAMP (qLAMP) experimental trends. The immunostaining results were consistent with previous literature reports and with our qLAMP and qPCR results. We present a proof-of-concept demonstration of the use of a LAMP-based POC platform for the identification or discrimination of cancer tissues. Our strategy can be extended to other diseases associated with altered gene expression in body tissues or fluids.

## 1. Introduction

Today, cancer remains a significant global health challenge, ranking as the second leading cause of death worldwide [1]. However, the burden of cancer is not equally distributed among our societies [1]. Indeed, the burden of cancer is rising in low-income and middle-income countries (LMICs), where approximately 65% of global cancer-related deaths occur annually [2]. Early diagnostics have been recognized as the most cost-effective strategy for reducing cancer burden [2,3,4]. Nevertheless, the diagnosis of cancer continues to be an enormous challenge, precisely in underdeveloped economies. Current detection strategies require highly trained specialists and sophisticated equipment. The use of specialized detection techniques increases overall healthcare costs and limits the availability of medical diagnosis, highlighting the need for easy-to-implement and cost-effective detection strategies to improve patient prognosis [5,6].

One strategy that can help address these challenges, especially in regions with limited health infrastructure, is to use point-of-care (POC) systems, which enable rapid and cost-effective on-site diagnosis [5,7,8,9]. POC diagnostic platforms generate cost reductions by replacing expensive, labor-intensive techniques and they enable economies in cancer treatment by identifying cancer at earlier stages. Furthermore, early detection through POC systems leads to better prognosis and reduces mortality rates. An early cancer diagnosis is crucial for reducing treatment costs and increasing patient survival rates because cancer treatment costs and treatment failures escalate with advancing disease stages. Locations that lack proper health infrastructure or have only limited access would benefit from POC systems by decreasing their dependence on central laboratories.

POC systems also represent a promising opportunity for early detection of cancer biomarkers in easily sampled bodily fluids, such as blood, tissue, or saliva [8,9,10]. For diseases such as cancer, which are characterized by the overexpression of tumor-specific or associated biomarkers, combining rapid and cost-effective POC technologies with high-accuracy testing for the detection of specific biomarkers could facilitate and enhance the diagnostic pipeline by indicating the presence and abundance of cancer cells in tissues or bodily fluids [11,12,13]. Of equal importance, the presence or absence of specific biomarkers also indicates the developmental stage of a cancer in a specific patient. Evaluation of these biomarkers may allow physicians to identify and discriminate between different cancer types and molecular subtypes, thereby providing access to proper treatment based on early and accurate diagnosis [14,15,16,17].

Cancer detection efforts toward affordable POC molecular diagnostics include paper-based microfluidics [18,19], smartphone-assisted fluorescence readers [20,21], portable, CRISPR-based detection systems [19,22], and electrochemical biosensors [23,24]. Recent studies have reported the determination of RNA (as a cancer biomarker) using different strategies assisted by nucleic acid amplifications [25,26,27], including isothermal amplification techniques [26,27].

The objective of this study was to develop and validate a simple, low-cost, Arduino-based real-time loop-mediated isothermal amplification (LAMP) platform for POC detection of cancer-associated biomarkers. This Arduino-based system can monitor (LAMP) reactions in real time to discriminate between different tissue spheroids derived from different cell types, including cancerous and non-cancerous human cells.

## 2. Materials and Methods

### 2.1. Overall Strategy

Recently, molecules related to gene expression, such as non-coding RNA (including microRNA [miRNA] [28,29] and long-noncoding RNA [lncRNA]) [30,31], have emerged as potential novel biomarkers for cancer diagnosis. These markers are aberrantly expressed in cancer patients and could conceivably be detected using more affordable genetic amplification strategies than the presently used sequencing or immunostaining techniques. The detection and quantification of genetic expression biomarkers might enable early cancer detection and even molecular subtype identification.

**Figure 1:**
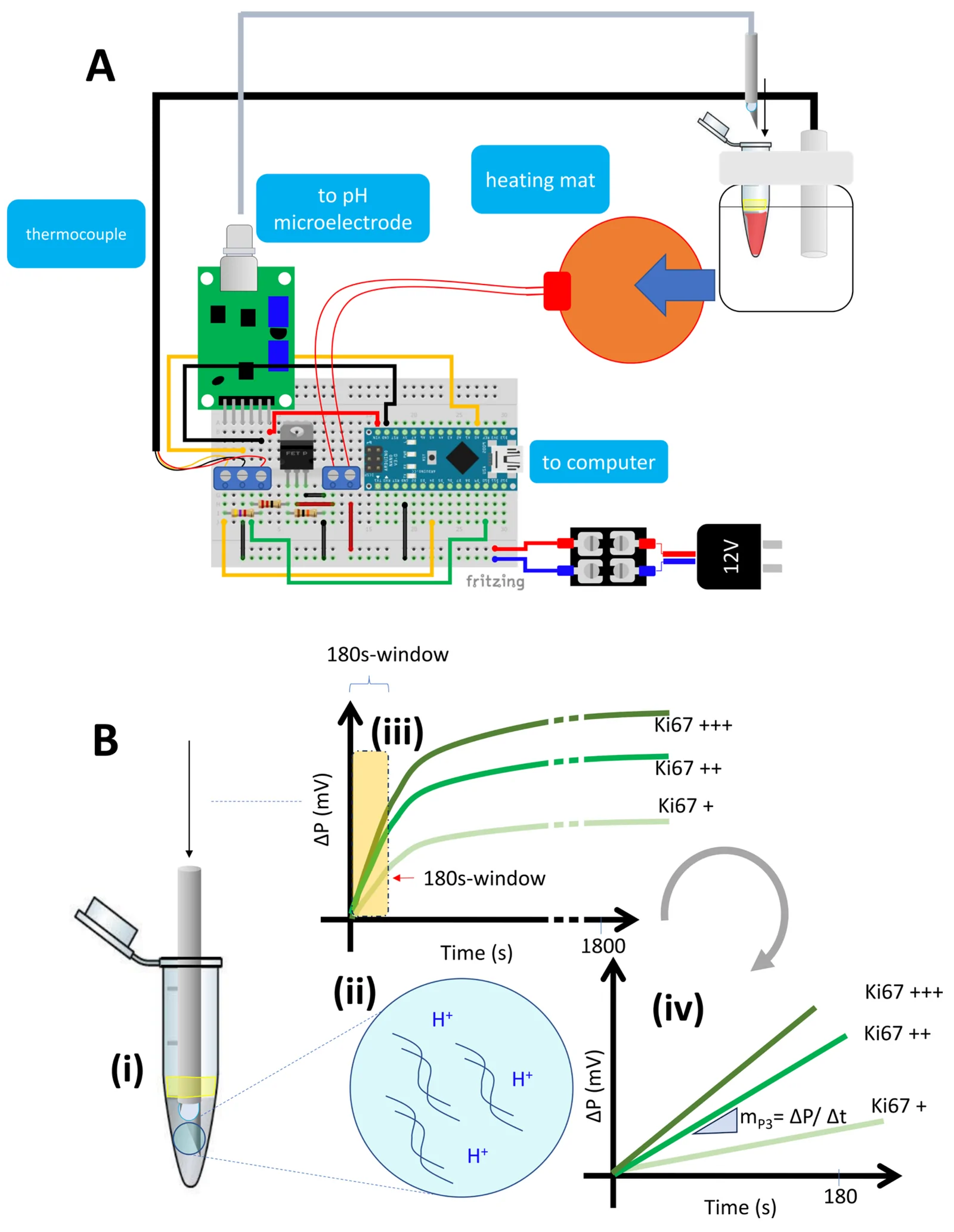
Experimental setup of the Arduino-based qLAMP system. (**A**) Schematic representation of the Arduino-based PID temperature controller and online electric potential monitoring system; a heating mat (indicated by the blue arrow) connected to an Arduino-based PID controller is used to control the temperature at 65 °C. (**B**) A microelectrode (i) inserted into the reaction mix monitors, in real time, the change in electrical potential due to (ii) the release of hydronium ions that occurs as the targeted amplification of RNA takes place. (iii)The change in the electrical potential (ΔP) during the first 3 min of the amplification (indicated by the yellow shade) is particularly rapid, and (iv) the plot of ΔP versus time renders a straight line with a slope (m_P3_) that is proportional to the amount of target RNA initially present in the reaction mix.

In this study, we used a Loop mediated Amplification (LAMP) strategy enabled by a portable Arduino-based device equipped with a pH microelectrode (Figure 1) to determine the expression of four relevant cancer biomarkers (estrogen receptor, progesterone receptor, Ki67, and Her2) and a housekeeping gene (*GAPDH*) in spheroids fabricated from four different human cell lines: a human breast cancer cell line (MCF7), a triple-negative breast cancer cell line (MDA-MB-231), a human prostate cancer cell line (Du145), and a non-cancerous fibroblast cell line (BJ). The overall aim of these amplification experiments was to demonstrate that this LAMP-based strategy can effectively discriminate tissues derived from these cell lines. The results from these LAMP-based amplification experiments were compared to conventional qPCR and immunostaining results.

### 2.2. LAMP Reaction & m_P3_ Detection Strategy

Figure 1 shows a scheme of the Arduino-based quantitative LAMP (qLAMP) system, similar to that reported by Alvarez et al. (2021) [32], which is capable of isothermal incubation and real-time online monitoring of the LAMP reaction, facilitated by a pH microelectrode. Figure S1 A shows an image of the complete qLAMP system. The system consists of three main components: the pH microelectrode connected to the Arduino, a Dallas thermocouple sensor for real-time temperature monitoring, and a heating mat to maintain the proper temperature for the LAMP reaction (Figure 1A). Our amplification experiments require isothermal heating of samples at 65 ± 1.5 °C. Using this Arduino-based prototype, we monitored the electrical potential progression in the reaction mix online during amplification. All reactions were conducted in a final reaction volume of 50 μL within standard 200 μL PCR-Eppendorf tubes. Each tube contained 25.0 μL of commercial WarmStart^®^ Colorimetric LAMP 2× Master Mix (DNA & RNA) from New England Biolabs (Boston, MA, USA) and 14.2 μL of nuclease-free water, to achieve a final reaction volume of 39.2 μL.

Separately, we prepared 8.8 μL of primer solution, consisting of specific concentrations of six primers (FIP, BIP, F3, B3, LoopF, and LoopB).

For each amplification experiment, we added 2 μL of RNA sample extracted from tissue spheroids to the Eppendorf tube containing the reaction mix diluted with nuclease-free water. Subsequently, 8.8 μL of primer mix was introduced. We then inserted the microelectrode tip into the reaction mix and added a drop of mineral oil to prevent evaporation. After each LAMP reaction experiment, we cleaned the pH microelectrode tip to eliminate any residual genetic material that could interfere with subsequent reactions. To do this, we rinsed the microelectrode tip thoroughly with PBS and immersed it in a 25% Clorox solution (1 part Clorox, 3 parts PBS) for 15 min at 65 °C [32,33].

We assessed the initial LAMP trajectory by monitoring the change in electrical potential (ΔP) during the first 3 min of the reaction, starting from the addition of the primer mix. Here, ΔP = P(t) − P_0_, where P_0_ is the electrical potential at the beginning of amplification and P(t) is the electrical potential at time t. During this initial 3-min interval, ΔP exhibited an approximately linear dependence on time. We therefore defined m_P3_ as the slope of the linear fit of ΔP versus time over the first 180 s of the reaction, providing an indicator of the initial amplification rate.

### 2.3. Primer Design

Independent sets of primers, each composed of six primer sequences (*FIP*, *BIP*, *F3*, *B3*, *LoopF*, and *LoopB*), were developed for each of the five examined genes (*ESR 1*, *PGR*, *Ki67*, *Her2*, and *GAPDH*). Table 1 shows the sequence of each of the primers used in this work.

### 2.4. Cell Culture and Spheroid Fabrication

We fabricated tissue spheroids derived from the four commercial cell lines to validate our diagnostic strategy. Spheroids were fabricated using conventional strategies [34,35] based on the use of ultra-low adherence 96-well plates. For the fabrication of tissue spheroids, we used all four cell lines (MCF7, HTB-22™; MDA-MB-231, HTB-26™; DU145, HTB-81™; and BJ, CRL-2522™), obtained from the American Type Culture Collection (ATCC, Manassas, VA, USA). For cell culture, we employed Minimum Essential Medium Eagle (EMEM) (Sigma-Aldrich, St. Louis, MO, USA), Dulbecco’s Modified Eagle’s Medium (DMEM), fetal bovine serum (FBS) (Gibco, Grand Island, NY, USA), an antibiotic-antimycotic (Anti-Anti) (Gibco, Grand Island, NY, USA), and 0.25% trypsin-EDTA (1X) (Gibco, Grand Island, NY, USA).

We initially cultured the cells in T25 flasks, followed by T75 flasks, in a humidified environment with 5% CO_2_ at 37 °C. Subsequently, we transferred the cells to 96-well Ultra-Low-Attachment (ULA) plates for spheroid formation. We cultivated the MCF7, Du145, and BJ cell lines using EMEM supplemented with 10% FBS and 1% antibiotic-antimycotic. The MDA-MB-231 cells were cultured in DMEM supplemented with 10% FBS and 1% antibiotic-antimycotic. We monitored the culture medium daily and replaced it with fresh medium every 48 h. When cells reached 70–80% confluence in a T25 flask, we detached them using 0.25% Trypsin-EDTA (1X). We then separated the cells by centrifugation (5 min at 1000 rpm) and cultured them again in a T75 flask containing 8 mL of culture medium. We fabricated an average of 24 spheroids from each cell line using 96-well ULA plates and seeding 5 × 10^4^ cells in each well in a volume of 200 μL. After a 72-h incubation period, we used six sets of 20 spheroids (~1 × 10^6^ cells spheroid^−1^) for RNA extraction and 4 spheroids for immunostaining (one spheroid per biomarker). We performed RNA extraction using the RNeasy Plus Mini Kit (QIAGEN, Germantown, MD, USA). From each extraction, we obtained nearly 50 μL of genetic material (RNA). For each cell line, spheroids were fabricated under identical conditions within the same experimental batch. Each RNA preparation subsequently underwent three independent qLAMP and three RT-qPCR analyses. Therefore, the reported replicates correspond to independent RNA extractions obtained from separate spheroid populations generated within the same fabrication batch.

### 2.5. RT-qPCR Experiments for Biomarker Analysis

RT-qPCR experiments were conducted using Qiagen’s QuantiNova SYBR Green RT-PCR Kit in a Rotorgene Q 5plex HRM real-time thermal cycler at a final volume of 20 μL according to the manufacturer’s instructions. Separately, we prepared a primer mix at a concentration of 0.5 μM, which included LAMP primers *F3* and *B3* (Table S1). For each replicate, a 1 μL aliquot of RNA extracted from spheroids (1 × 10^6^ cells) was added to each reaction. Subsequently, 1 μL of the primer mix was introduced to each reaction tube. The assembled reaction mixtures were subjected to amplification for 40 cycles under the conditions recommended by the manufacturer. We applied the ΔCt method for data analysis. This method involved subtracting the Ct value of the housekeeping gene from the Ct value of the gene of interest to provide a normalized measure of gene expression. Relative quantification of gene expression was performed using the 2^(−ΔCt)^ method.

### 2.6. Principal Component Analysis & Hierarchical Clustering Analysis

We sequentially used principal component analysis (PCA) [36,37] and clustering analysis [38] to identify relationships and clusters within the data sets derived from the qLAMP or qPCR experiments. Based on PCA, as conducted using Python software (Python 3.x (Python Software Foundation, Wilmington, DE, USA) and the scikit-learn 1.8.0 library, we generated a score to visually identify the two variables (i.e., components) that most contribute to the variance of data (m_P3_ values or log_2_(2^−ΔCt^) for the analysis of qLAMP or qPCR derived results, respectively).

Principal component analysis (PCA) [36,37] was performed independently for the qLAMP and RT-qPCR datasets using GAPDH-normalized gene expression values for each biological replicate. For qLAMP, three replicate measurements of the calculated m_P3_ values were considered for each cell line and each evaluated biomarker (*ESR1*, *Her2*, *Ki67*, and *PGR*). For each biological replicate, the m_P3_ value obtained for each biomarker was normalized to the m_P3_ value obtained for GAPDH from the corresponding biological replicate. The resulting multivariate data matrix therefore contained replicate-specific *GAPDH*-normalized expression values, with each row corresponding to one biological replicate and each column to one evaluated biomarker. To explore the effect of progressively increasing the number of biomarkers considered, PCA was performed sequentially using two (*ESR1* and *Ki67*), three (*ESR1*, *PGR*, and *Ki67*), and four (*ESR1*, *PGR*, *Ki67*, and *Her2*) biomarkers. For RT-qPCR, Ct values were normalized to the *GAPDH* housekeeping gene by calculating ΔCt = Ct_target_ − Ct_GAPDH_ for each biological replicate. Relative expression values were calculated as 2^−ΔCt^. For PCA, the corresponding log_2_-transformed relative-expression values, equivalent to −ΔCt, were used, thereby avoiding the strong skewness associated with exponentially transformed expression values. As for the qLAMP dataset, each row of the resulting matrix represented one biological replicate, and each column represented one of the four evaluated biomarkers (*ESR1*, *Her2*, *Ki67*, and *PGR*).

PCA was performed in Python using the scikit-learn library. For qLAMP, *GAPDH*-normalized m_P3_ values were analyzed without additional transformation; for RT-qPCR, the −ΔCt values described above were used. In all analyses, variables were mean-centered but not variance-standardized; consequently, PCA was performed based on the covariance structure of the data. Principal component scores were used to visualize similarities and differences among biological replicates, while loading vectors were examined to assess the relative contribution of individual biomarkers to the observed variance. PCA was treated exclusively as an unsupervised exploratory analysis and was not used to develop, train, or validate a supervised classification or predictive model.

Hierarchical clustering was performed independently for each set of normalized expression profiles in Python 3.13.5 using the SciPy library 1.17.0. Euclidean distance was used as the dissimilarity metric, and average linkage was used as the agglomeration method. For qLAMP, hierarchical clustering was performed independently for the same two-, three-, and four-biomarker datasets evaluated by PCA; for RT-qPCR, the four-biomarker dataset was analyzed. Clustering was performed directly on the corresponding normalized biomarker-expression vectors rather than on the PCA scores. No predefined dendrogram cut threshold was applied, and the resulting dendrograms were interpreted qualitatively as exploratory representations of the relationships among biological replicates.

### 2.7. Immunostaining Assays and Image Analysis

We immunoassayed spheroids derived from each cell line (MCF7, MDA-MB-231, Du145, and BJ) using four distinct primary antibodies, each targeting the *ESR 1* estrogen receptor, the *PGR* progesterone receptor, the Ki67 protein, or the *Her2* receptor. After 3 days of culture in ULA plates, we retrieved the spheroids and washed them with PBS for 5 min to remove adhering cell culture media. The spheroids were then immersed in 4% paraformaldehyde for 30 min at room temperature for cell fixation and washed three times with PBS to remove the remaining paraformaldehyde. The spheroids were treated with Triton X-100 solution (5%, *v*/*v* in PBS) for 45 min to permeabilize them and then washed twice with PBS (each wash for 5 min). We blocked non-specific adhesion sites by overnight incubation in a solution containing 5% bovine serum albumin (BSA), 0.3 M glycine, and 0.5% Triton X-100 at 4 °C.

We used Alexa Fluor^®^ conjugated antibodies for the immunostaining of *Her2* and *PGR*: an anti-Neu/ErbB2/Her2 antibody (3B5) Alexa Fluor^®^ 680 and an anti-progesterone antibody (11P14) Alexa Fluor^®^ 594 (Santa Cruz Biotechnology Inc., Dallas, TX, USA). Spheroids that had previously been fixed, washed, and blocked were incubated overnight in a 1:50 solution (in blocking solution) of the primary antibody at 4 °C.

For the identification of *Ki67* and *ESR 1*, we used an anti-*Ki67* antibody and an Anti-Estrogen antibody (Santa Cruz Biotechnology Inc., Dallas, TX, USA). In these assays, spheroids previously fixed, washed, and blocked were incubated overnight in a 1:150 solution (in blocking solution) of the primary antibody at 4 °C. After incubation, spheroids were washed three times with PBS (each wash for 7 min), treated with a 1:400 solution (in blocking solution) of a secondary antibody (donkey anti-mouse IgG H&L conjugated with Alexa Fluor 488, ab150105, Abcam, Boston, MA, USA), and incubated overnight at 4 °C. The spheroids were then washed twice with a washing solution (0.2% Triton X-100 and 0.04% Tween 20 in PBS).

In all cases, we counterstained the cell nuclei and cytoskeletons by immersion in a phalloidin solution (Phalloidin-iFluor 647 Reagent from Abcam, Boston, MA, USA; 2:1000 dilution in PBS) and a DAPI solution (2:1000 dilution of DAPI from Sigma-Aldrich, St. Louis, MO, USA; 2:1000 dilution in PBS) for 1 h at 37 °C. The samples were then washed twice with PBS (each wash for 10 min) and brightfield and fluorescence images were obtained using an Axio Observer.Z1 microscope (Zeiss, Oberkochen, Germany) equipped with Colibri.2 LED illumination and an Apotome.2 system (Zeiss, Oberkochen, Germany). We obtained merged micrographs using the microscope software (Zen Blue Edition, Zeiss, Oberkochen, Germany).

### 2.8. Statistical Analysis

All statistical analyses were performed using Minitab statistical software (version 21.4.1). All amplification and immunostaining assays were run (at least) as triplicate biological samples.

## 3. Results and Discussion

### 3.1. LAMP Assays: Discrimination Among Different Tissues

Here, we explored the use of this portable LAMP Arduino-based system for the diagnosis and discrimination of cancer types. We previously used this system to quantitatively discriminate between saliva samples from subjects infected or not infected with SARS-CoV2 [32]. The Arduino-based system enables the isothermal amplification of RNA extracted from tissue samples and the on-line measurement of the change in electrical potential during amplification using a microelectrode in Eppendorf tubes.

The principle underlying the specific RNA identification of this system is illustrated in Figure 1B and Figure S1. During LAMP, DNA polymerase incorporates deoxynucleoside triphosphates (dNTPs) into the growing DNA strand complementary to the target RNA-derived sequence. Each nucleotide incorporation reaction releases pyrophosphate and a proton (H^+^) into the reaction mix [39,40] as part of the polymerization process (Figure S1). Therefore, successful nucleic acid amplification results in the progressive accumulation of protons.

Under our weakly buffered reaction conditions, this electrical potential change is particularly pronounced during the initial stages of amplification, as we previously demonstrated for SARS-CoV-2 RNA detection [32]. Conveniently, ΔP exhibits an approximately linear dependence on time during the first 3 min of the reaction (Figure 1B). The slope of the ΔP versus time trajectory over this interval (m_P3_) is related to the amount of genetic material initially present in the reaction mix and therefore provides a quantitative indicator of the abundance of the specific target mRNA sequence in the sample.

In the following sections, we show that this LAMP-based strategy enables us to establish the presence of cancerous cells and to distinguish between cancerous and non-cancerous tissues and between different types of cancerous tissues. This discrimination was based on the determination of the relative level of expression of a set of cancer-associated genes (in this case, four genes) in each cell line with respect to a housekeeping gene.

In this study, we chose four biomarker genes relevant to breast cancer: the estrogen receptor alpha (*ESR 1*), the progesterone receptor (*PGR*), the proliferation marker *Ki67*, and the human endothelial growth factor receptor (*Her2*). We also selected glyceraldehyde 3-phosphate dehydrogenase (*GAPDH*) as the housekeeping gene.

These selections were based on the relevance of these genetic expression biomarkers among different types of cancer. In brief, estrogen (and the estrogen receptor) are involved in cancer initiation and progression. Furthermore, *ESR 1* interacts with endothelial-to-mesenchymal transition regulators, resulting in tumor invasion [41,42,43]. Regarding breast cancer hallmarks, the progesterone receptor is another member of hormonal receptor families. The activation of the progesterone receptor suggests active *ESR* signaling. *PGR*-positive tumors are rarely estrogen receptor negative (0.2–10%). PGR expression is commonly used alongside estrogen receptor expression in BRCA tumor subtyping [41,44]. The double-positive cancer group that shows activation of both estrogen and progesterone receptors accounts for up to 55–65% of cases of breast tumors [41,44,45,46,47]. Ki67 is a nuclear protein that is considered a proliferation marker, as its expression peaks during mitosis. High Ki67 expression levels are correlated with cancer metastasis and recurrence; thus, Ki67 is also a prognostic and molecular subtype biomarker [48,49,50]. The role of *Her2* in tumorigenesis involves its ability to auto-phosphorylate tyrosine residues, in turn leading to the triggering of signaling pathways associated with cancer development and proliferation [51,52]. Finally, *GAPDH* is a frequently used housekeeping gene in gene expression studies, based on the assumption that the expression levels of its associated mRNA are practically constant within the same tissue. However, different tissue types may exhibit significantly different expression levels of *GAPDH* [53].

**Figure 2:**
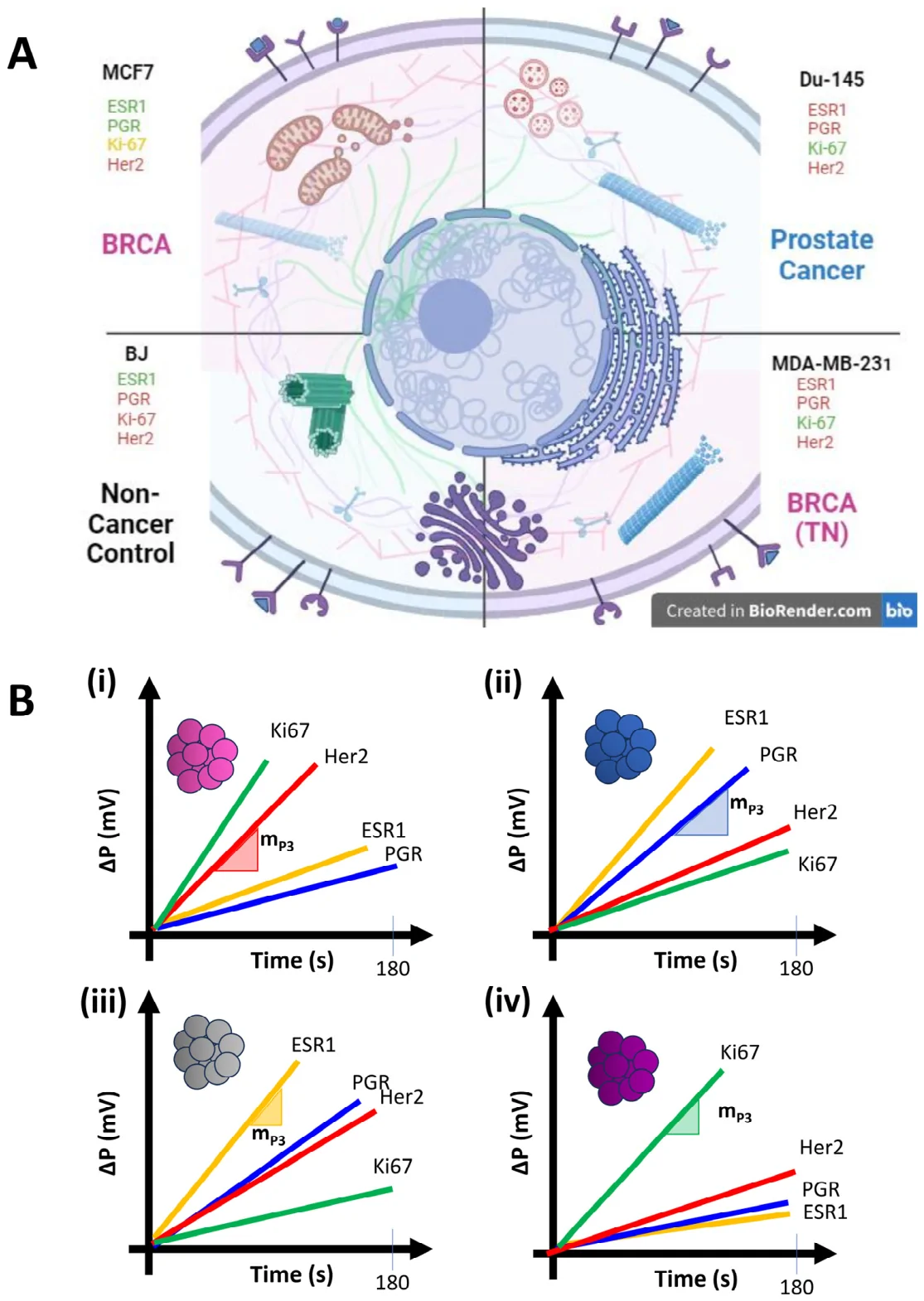
Expected expression profiles for selected genetic biomarkers in different cell lines. (**A**) Schematic representation of the 4 different cell lines (MCF7, Du-145, BJ, and MDA-MB-231) used in amplification experiments of 4 biomarkers (*ESR 1*, *PGR*, *Her2*, and *Ki67*). The expression levels of biomarkers relevant to cancer physiology are also represented. Biomarkers labeled in green are overexpressed in that cell line, red labels signify underexpression, while yellow indicates near normal expression. (**B**) Since the set of m_P3_ values associated with the amplification of *ESR 1* (yellow lines), *PGR* (blue lines), *Her2* (red lines), and *Ki67* (green lines), is expected to differ in each cell line, the discrimination between these four lines is feasible based on analysis of the m_P3_ value sets.

We also selected four different cell lines (MCF7, MDA-MB-231, DU145, and BJ cells) and fabricated tissue spheroids from each. The rationale behind this selection was to include cell lines representing different types of cancer (MCF7 and MDA-MB-231 cells for breast cancers and DU145 cells for prostate cancer) or healthy tissue (in this case, BJs) to explore a range of different expression profiles. In our experiments, MCF7 cells represent the breast cancer Luminal A molecular subtype, which is characterized by positive expression of both *ESR* and *PGR*, absence of *Her2*, and low levels of *Ki67* [54,55] (See also Figure 2A). The MDA-MB-231 cell line exhibits an expression profile that lacks expression of the hormonal receptors (*ESR* and *PGR*) and *Her2*. This cancer cell line is considered highly aggressive and invasive [56]. Du145 is an androgen-independent prostate cancer cell line that expresses lower levels of *ESR* and *PGR* than other human prostate cancers [57,58,59]. The basal expression of *Her2* in Du145 cells is expected to be higher than their *ESR* expression [60]. BJ fibroblasts are derived from non-cancerous foreskin tissue of a newborn. The expression levels of *Her2* and *Ki67* markers are expected to remain low, indicating the absence of tumor development. Since the expression profiles of the chosen biomarkers varied between the selected cell lines, we hypothesized that our qLAMP system should be able to discriminate among these lines (see Figure 2A). We hypothesized that the slope values (m_P3_), as quantitative indicators of the expression of the selected biomarker, would vary significantly among the pool of selected biomarkers (Figure 2B). Consequently, by obtaining an expression profile (a set of m_P3_ values) for each cell line, we might discriminate between them using subsequent statistical analyses and/or principal component analysis (PCA) and hierarchical clustering.

**Figure 3:**
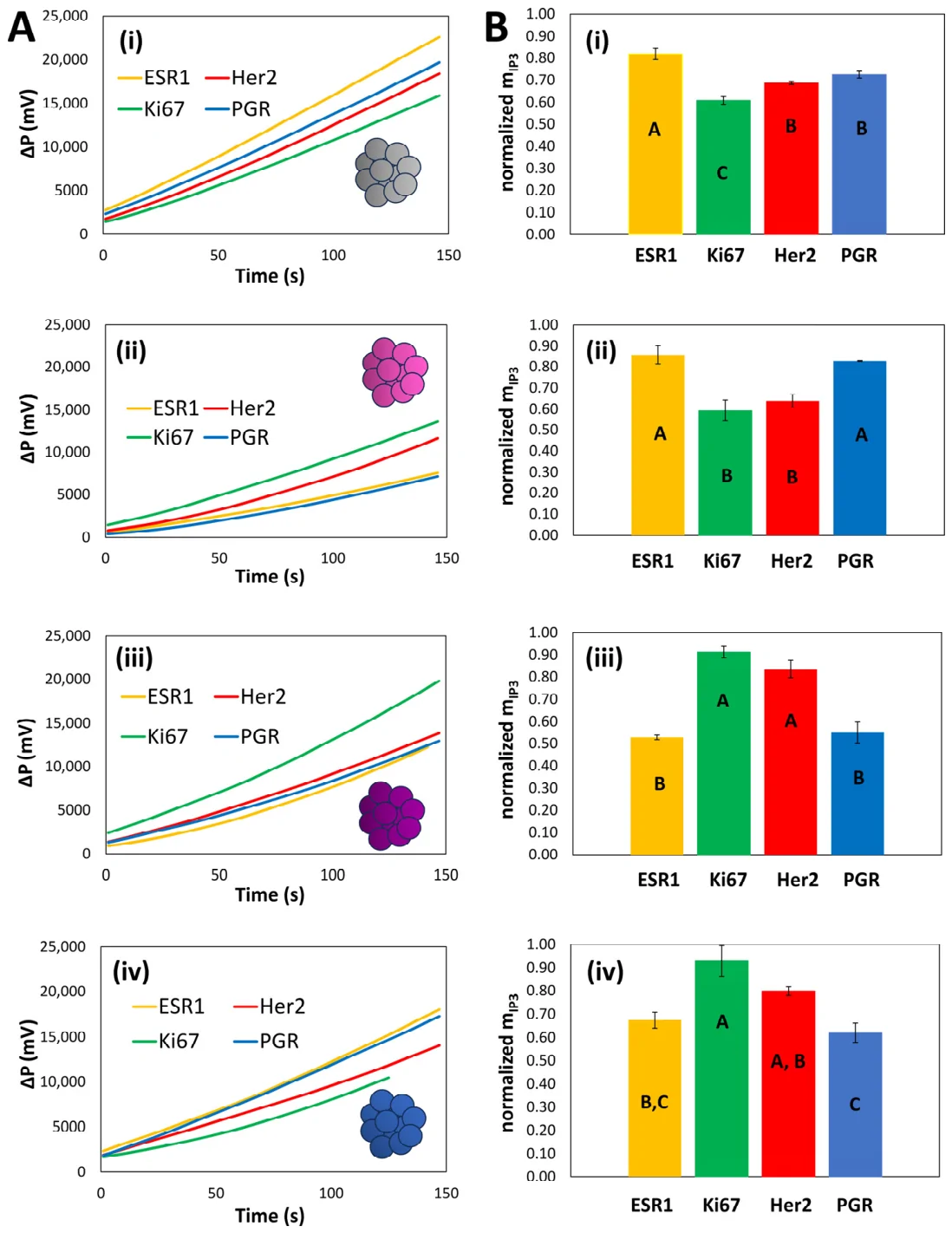
Experimental LAMP expression profiles for selected genetic biomarkers in different cell lines. (**A**) Sets of LAMP-derived m_P3_ values for relevant cancer biomarker genes (*ESR 1*, *PGR*, *Her2*, and *Ki67*) for microtissues derived from four different cell lines (MCF7, Du-145, BJ, and MDA-MB-231 cells). (**B**) Sets of normalized m_P3_ values for spheroid tissues derived from the four different cell lines. Groups with different letters are significantly statistically different from each other (Confidence level of 95% (α = 5.0%)). For each biological replicate, biomarker-specific m_P3_ values were normalized to the *GAPDH* m_P3_ value obtained from the corresponding replicate and color coded as follows: *ESR 1* (yellow), *PGR* (blue); *Her2* (red) and *Ki67* (green).

Indeed, our proof-of-principle results demonstrate that this LAMP strategy can successfully distinguish tissue spheroids fabricated from the four selected commercial cell lines (three cancer cell lines and a healthy tissue cell line). The set of m_P3_ values, obtained for each of the expression biomarkers and cell types included in this study, is presented in Figure 3A. These m_P3_ values were also normalized by dividing the m_P3_ value of the *GAPDH* gene (i.e., the selected housekeeping gene) (Figure 3B).

As shown in Figure 3B, we identified significant statistical differences in the expression of the gene biomarkers within the same cell line. Notably, obvious differences were evident in the profile of m_P3_ values among the different spheroid tissues.

As a result, it was possible to discriminate the genetic expression across spheroid types using the selected biomarkers. For example, we found clear differences in the expression of markers between the MCF7 and MDA-MB-231 breast cancer cell lines. As anticipated, we observed higher expressions of the *ESR 1* and *PGR* markers in the MCF7 cell line than in the MDA-MB-231 cell line (please compare Figure 2A and Figure 3A,B). Also, as expected, the MDA-MB-231 spheroids (triple-negative breast cancer) displayed an overexpression of the Ki67 proliferation marker. In prostate cancer spheroids derived from the Du145 cell line, the expression levels of the proliferation gene *Ki67* were higher than the expression levels of *ESR 1* and *PGR* (please compare Figure 2A and Figure 3A,B). Indeed, the differences in gene expression among the cell lines are significant (see also Table 2) and could enable clear discrimination between pairs of cell lines. For instance, the expression level of *ESR 1* could be used to differentiate clearly between MCF7 and MDA-MB-231 spheroids (Table 2). Similarly, the high expression of *Ki67* in MDA cells could also be used to distinguish between tissues derived from MDA-MB-231 and MCF7 cells. However, some indicators cannot be used alone to discriminate between cell lines. This is the case for *Her2*, which seems to exhibit similar levels of expression among different cell types. While the overexpression or underexpression of a single gene can help to distinguish between pairs of cell lines (Table 2), the use of a set of indicators should be a more robust strategy to provide conclusive discrimination between tissue types.

### 3.2. Clustering Analysis

We also pursued quantitative discrimination and identification cell type clusters via their expression profiles using principal component analysis and a simple hierarchical clustering algorithm.

PCA was performed as an unsupervised exploratory analysis to investigate whether the normalized expression profiles (i.e., sets of normalized m_P3_ values) generated by qLAMP exhibited natural grouping patterns among the evaluated cell lines.

**Figure 4:**
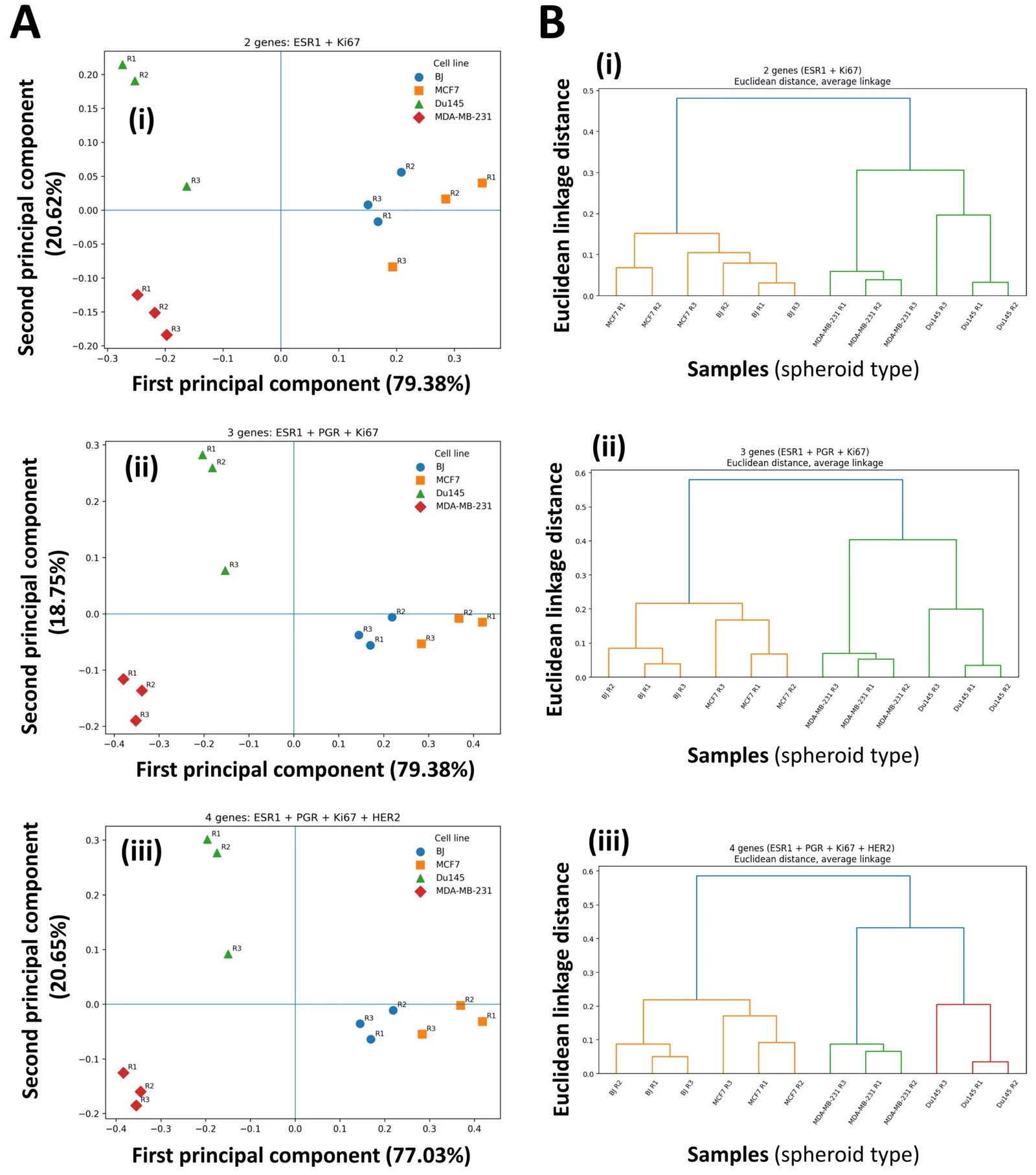
Principal component analysis (PCA) and clustering of the mP3 values derived from LAMP assays. (**A**) PCA score plot illustrating the unsupervised distribution of samples according to their normalized gene-expression profiles. Cell lines are color coded as follows: MCF7 (orange), BJ (blue), MDA-MD-231 (red), and DU-145 (green). (**B**) clustering dendrogram for the m_P3_ set values corresponding to the expression of (i) two genes (*ESR 1* and *Ki67*); (ii) three genes (*ESR 1*, *Ki67*, and *PGR*); and (iii) four genes (*ESR 1*, *Ki67*, *PGR*, and *Her2*), as determined from LAMP assays conducted using spheroids derived from 4 different cell lines. See also Table S2.

Figure 4A(i) shows a PCA score that considered the m_P3_ values of the *ESR 1* and the *Ki67* genes only. The analysis chooses the two main components of the variance of the data (in this case, the m_P3_ values derived from the amplification of the *ESR 1* and the *Ki67* genes) and plots the variance of each data point in a 2D plot. Visually, the data points corresponding to BJ and MCF7 spheroids are in close proximity. An analysis of the Euclidean distances between the points in Figure 4A(i) reveals the existence of 4 clusters, as also shown in the dendrogram in Figure 4B(i). However, one BJ replicate clustered together with the MCF7 observations. When the m_P3_ values of three genes (i.e., *ESR 1*, *PGR*, and *Ki67*) were included in the PCA, four clusters of points were distinguishable (Figure 4A(ii)) and the classification algorithm, based on the Euclidean distance between points, correctly placed all points in clusters that contained only one type of spheroid (Figure 4B(ii)). Similarly, when all genes (*ESR 1*, *PGR*, *Ki67*, and *Her2*) were included in the PCA, four clusters were clearly observed (Figure 4A(iii)), and the resulting clusters corresponded to the four cell-line groups, with replicates from each cell line clustering together. (Figure 4B(iii)). The PCA findings suggest that the set of biomarker genes selected in this study is sufficient to clearly discriminate between the four types of tissues studied here. Note that this simple classification algorithm is fully unsupervised. Other than the data points, no additional information is provided. The clustering decisions are solely guided by the variance of the data sets provided. Note that in this case (when the full set of m_P3_ values was considered), the first two principal components accounted for 75.3% and 20.4% of the total variance, respectively, explaining 95.7% of the overall variability in the dataset. This high cumulative variance indicates that the two-dimensional PCA representation captures most of the multivariate structure of the data (Figure 4A(iii); Table S2). Examination of the loading vectors (Supplemental Table S1) revealed that PC1 was primarily influenced by *ESR1* and *PGR* expression, whereas PC2 was dominated by *Ki67*, indicating that differences in hormone receptor status and proliferative activity were the principal contributors to the observed separation among cell lines.

Conceptually, the ability to clearly discriminate between a set of different tissues would be further enhanced by considering a larger number of genes. Therefore, the proof-of-concept diagnostic strategy introduced here can be improved by selecting a larger set of biomarkers to create a more robust expression profile. Moreover, specific biomarkers known to be overexpressed in specific cell types will strengthen the robustness of the identification in specific tissues.

### 3.3. Comparison of LF-qLAMP and qPCR Expression Profiles

So far, our results show that it is feasible to discriminate between tissues derived from four different cell lines using portable qLAMP. Next, we compared our LAMP-based strategy with RT-qPCR—the current gold standard in molecular diagnostics (at least for infectious diseases) and a method widely used to assess gene expression.

**Figure 5:**
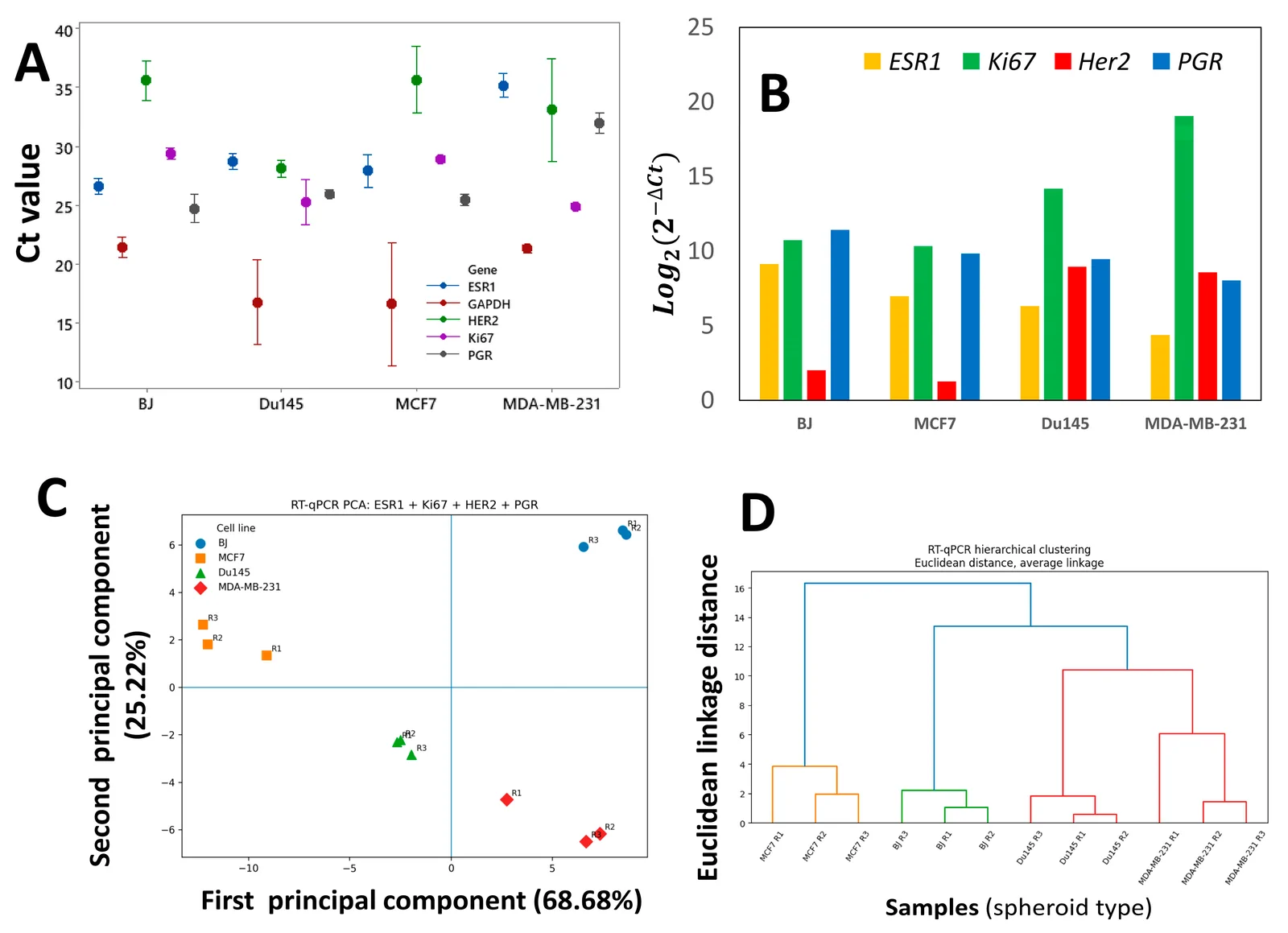
Gene expression determined by RT-qPCR assays. (**A**) Interval Plot of Ct Values; selected genes are color coded as follows: *ESR 1* (blue); *PGR* (gray); *Ki67* (purple), *Her2* (green), and *GAPDH* (red). (**B**) Comparison of log_2_(2^−ΔCt^) values for relevant cancer-related genes in 4 different cell lines. Genes are color coded as follows: *ESR 1* (yellow); *PGR* (blue); *Ki67* (green), *Her2* (red). The expression of each gene in each spheroid type was normalized against the average Ct value of the housekeeping gene (*GAPDH*). (**C**) PCA score plot illustrates the unsupervised distribution of samples according to their normalized gene-expression profiles. Cells are color coded as follows: BJ (blue), MCF7 (orange), DU145 (green), and MDA-MB-231 (red). (**D**) Dendrogram associated with the expression of 4 different genes, as measured by qPCR in spheroids derived from four different cell lines. See also Table S2.

Using a commercial qPCR thermocycler, we quantified the relative gene expression of *ESR 1*, *Her2*, *Ki67*, and *PGR* across the four cell lines (BJ, Du145, MCF7, and MDA-MB-231) used in our experiments. For each gene, we selected a primer set within the same sequence region targeted by our LAMP primer (see Table S1). In Figure 5A, we show Ct values for each cell line, color-coded by gene, and Figure 5B presents the log_2_(2^−ΔCt^) =−ΔCt values. As before, we used *GAPDH* as a housekeeping gene for normalization in Figure 5B, as its specific expression (expression level per unit of cellular mass) is expected to remain approximately constant across different experiments within the same cell line if the culture conditions are kept the same. We found significant differences in gene expression among the cell lines. Our findings are mostly in line with the literature and are also consistent with the results derived from the LAMP experiments (Figure 3). We observed a higher expression of the *ESR 1* gene in MCF7 cells (a hormone-dependent cell line) than in MDA-MB-231 cells. In agreement with the LAMP assays, the expression of *Ki67*, an indicator of proliferation, is significantly higher in MDA-MB-231 cells than in MCF7 cells. Similarly, Du145 cells (a cell line derived from a hormone-independent prostate cancer) exhibited significantly higher expression of the *Ki67* gene than the *ESR 1* and *PGR* genes, again consistent with the LAMP-based results (Figure 3A,B). However, we also observed some discrepancies between the LAMP and PCR results for specific genes and cell lines. For instance, the normalized expression of the *PGR* gene in MCF7 cells (a cell derived from a hormone-dependent breast cancer) was not much higher than that observed in MDA-MB-231 cells.

Figure 5C,D shows results of the PCA and clustering analysis derived from the qPCR results. As before, four clusters are observed. However, note that the distinction between BJ-derived and MCF7-derived spheroids is not visually clear for our selection of biomarkers and cell lines. However, a further application of clustering techniques based on the distance between the points in Figure 5C enables a discrimination between BJ and MCF7 cell types (Figure 5D; see also Table S2). Overall, both the qLAMP and qPCR strategies used in this study enable the discrimination of the four cell lines included in this study. However, discrepancies are observed in both techniques.

### 3.4. Comparison of LAMP Expression Profiles and Immunostaining Assay Results

**Figure 6:**
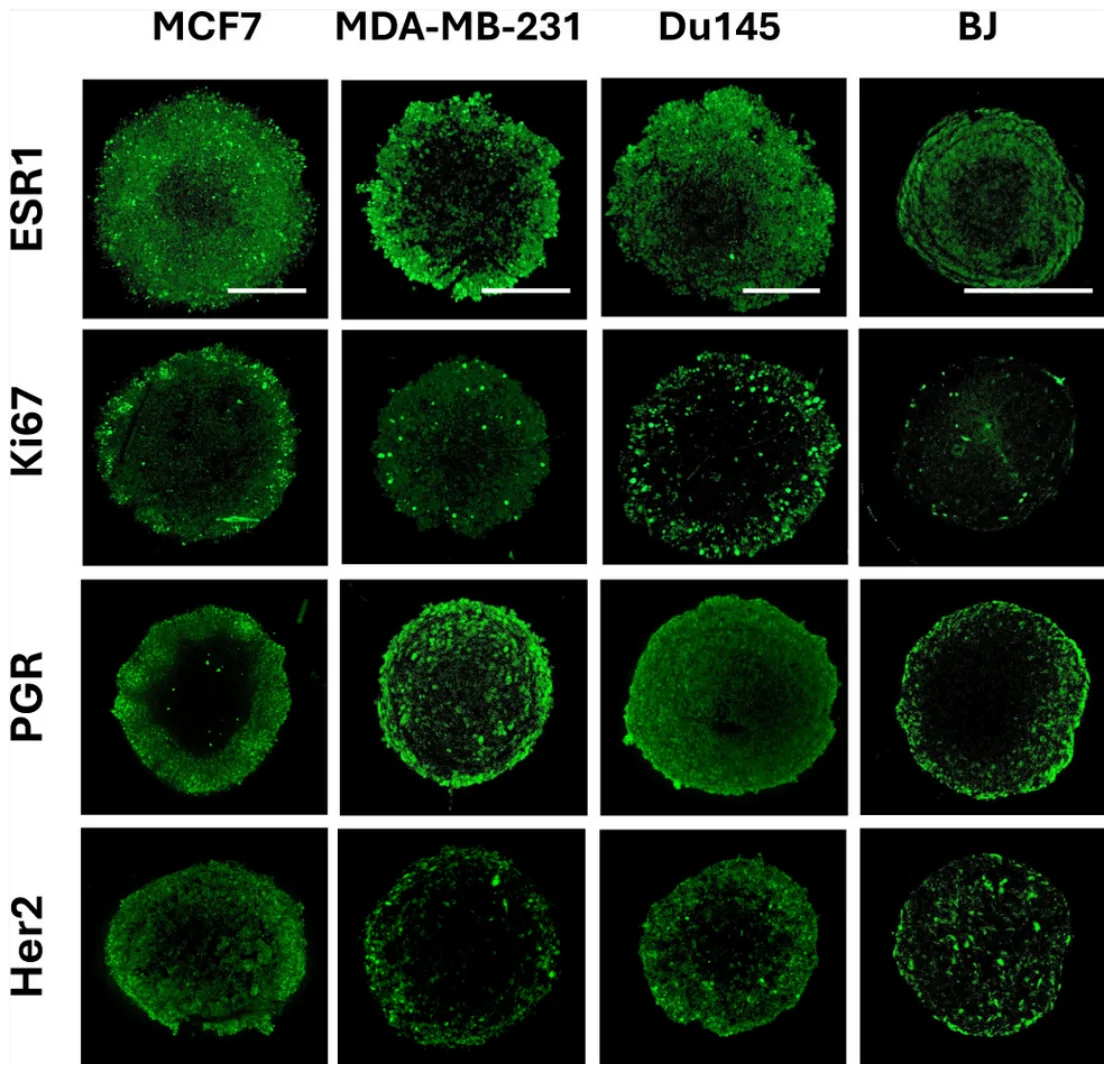
Immunostaining images of tissue spheroids derived from four different cell lines. Immunostaining assays reveal the presence of four relevant cancer biomarkers (*ESR 1*, *PGR*, *Ki67*, and *Her2*) in tissue spheroids fabricated from four different cell lines (MAC7, BJ, MDA-MB-231, and DU-145 cells). Biomarkers are marked with specific antibodies and exhibit green fluorescence by optical fluorescence microscopy. (Scale bar: 300 μm).

We also conducted immunostaining experiments to confirm the presence of the four selected biomarkers (*ESR 1*, *PGR*, *Ki67*, and *Her2*) in spheroids derived from the four selected cell lines (BJ, Du145, MCF7, and MDA-MB-231) (Figure 6). These experiments provided additional information to corroborate/validate the results of qLAMP and qPCR, allowing the visual identification of differences related to the relative presence of these biomarkers in different cell lines. In addition, these immunostaining assays provided information on the spatial distribution of specific biomarkers within the spheroid. For instance, as anticipated, the expression level is higher in the external layers of the spheroids than in the core.

In general, the immunostaining results were consistent with the expected results based on the literature. For example, the expression of the *ESR 1* and *PGR* genes was much higher in MCF7 than in MDA-MB-231 spheroids. In Du145 spheroids, the expression of the proliferation marker Ki67 was higher than the expression of the *ESR 1* and *PGR* genes. This is also consistent with the qLAMP and qPCR results discussed previously. In general, the expression level of *Ki67* was much higher in cancerous spheroids than in healthy BJ spheroids. Among the cancerous spheroids, the expression of *Ki67* was higher in MDA-MB-231 than in MCF7 and Du145 cells, which was consistent with the results of the qLAMP and qPCR determinations.

While most of the observations derived from the immunostaining assays are consistent with literature and with the determinations of gene expression by qLAMP and qPCR, we also found discrepancies. For example, the progesterone receptor seems to be highly expressed in the Du145 cell line, and the immunostaining results suggest a higher expression of the *PGR* and *ESR 1* genes than of the *Ki67* gene in Du145 spheroids. By contrast, the qLAMP and qPCR results suggest a lower expression of the *PGR* and *ESR 1* genes than of *Ki67* in the Du145 spheroids. Notably, immunostaining reveals high expression of *Her2* in MCF7 and Du145 spheroids, whereas the qLAMp determinations suggest that *Her2* is expressed at similar levels among the four cell lines studied, and qPCR results suggest that *Her2* is expressed at higher levels in Du145 and MDA spheroids than in MCF7 and BJ spheroids.

The present study constitutes a proof-of-concept validation performed using spheroids generated from well-characterized commercial cell lines. This experimental design enabled reproducible evaluation of the analytical capabilities of the proposed qLAMP platform under controlled conditions. However, cancer spheroids do not capture the biological complexity of clinical specimens. Patient-derived tissues frequently exhibit cellular heterogeneity, stromal and immune cell infiltration, variable RNA quality, and additional sources of biological and technical variability that were intentionally excluded from the present study. Further work should evaluate the performance of this platform using primary tissues, biopsies, and clinically relevant specimens before its implementation as a diagnostic technology.

## 4. Conclusions

Here, we introduced a platform for cancer discrimination based on an isothermal amplification (LAMP) and a portable Arduino-based device equipped with a pH microelectrode. The principle underlying the functioning of this platform is straightforward. During any nucleic acid amplification, hydronium ions are released into the reaction mix. In unbuffered conditions, this release leads to a change in pH and electrical potential. The Arduino-based system evaluates the change in the electrical potential (ΔP) during the first 3 min of the LAMP reaction using a pH microelectrode immersed in the amplification reaction mix. The plot of ΔP versus time (during the first 3 min of amplification) renders a straight line whose slope (i.e., the m_P3_ value) is a quantitative indicator of the expression of the gene targeted by the LAMP primers.

To illustrate the application of this strategy, we adapted an Arduino-based system previously used for the quantitative identification of SARS-CoV-2 in saliva samples. In the context of detection of COVID in saliva, our LAMP-based strategy targeted one of the genes encoding the production of the spike protein (S protein) of the SARS-CoV-2. For cancer discrimination, we developed five sets of ad hoc LAMP primers to quantify the expression of four relevant cancer genes (*ESR 1*, *PGR*, *Her2*, and *Ki67*) and a housekeeping gene (*GAPDH*). We also fabricated tissue spheroids derived from three commercial cancer cell lines and one normal fibroblast cell line for use as tissue testing models for the proposed diagnostic strategy.

Using the LAMP-based diagnostic strategy for the selected set of target genes, different “expression signatures” (i.e., sets of m_P3_ values) were consistently determined for each of the four cell lines. Therefore, discrimination between cancer cell lines was feasible based on their gene expressions. The expression signature observed for each tissue type was also consistent with the anticipated signature based on information reported in the literature. MCF7 cell line presents an expression profile in which *ESR 1*, and *PGR* are highly expressed compared to *Ki67* and *Her2*. We expected this result, since MCF7 is considered a Luminal A molecular subtype. By contrast, MDA-MB-231 exhibited a higher expression of the *Ki67* proliferation marker, which is suggestive of a higher invasion potential and aggressiveness than that exhibited by MCF7 cells. Du145 is considered an androgen-independent prostate cancer cell line. Consistently, we observed a higher expression of the *Ki67* proliferation gene than the *ESR 1* or *PGR* genes. Lastly, BJ presents a higher expression of the hormonal receptors (*ESR 1* and *PGR*) than the *Her2* and *Ki67* genes.

We used PCA and clustering analysis to identify clusters of experimental data points. PCA and clustering suggested that a set of three or four biomarkers (from the ones used in our experiments) was sufficient for clear discrimination between tissues derived from different cell lines. When the amplification results for all four target genes (*ESR 1*, *PGR*, *Ki67*, and *Her2*) or three genes (i.e., *ESR 1*, *PGR*, *Ki67*) were included in the PCA, four clusters of data points were clearly distinguishable; each set accurately grouped the amplification results of each spheroid type. Similarly, the clustering algorithm also identified four distinct groups, each corresponding to a different cell line. However, when a set of two genes was used (i.e., *ESR 1* and *Ki67*) clear discrimination was only achieved using the clustering algorithm.

We also ran quantitative PCR and immunostaining assays to confirm the results derived from our qLAMP strategy. The trends in expression determined by qLAMP and qPCR were consistent among the four types of tissues included in our study. The qLAMP and qPCR results were also consistent with the observations derived from additional immunostaining assays.

In this study, we selected a set of four markers commonly used for breast cancer research to discriminate between spheroids derived from fibroblasts, two types of breast cancer cells, and a prostate cancer cell line. Our results suggest that, for the set of biomarkers and cell types included in the study, four biomarkers were sufficient to accurately discriminate between the four tissue types included in the study. However, the number of biomarkers used could be expanded to enhance detection efficacy.

The nature of the biomarkers used should also be adapted for each application, as the inclusion of biomarkers relevant to each specific cancer type is expected to increase the accuracy of the differentiation between tissue types. Our results must be interpreted as a first proof-of-concept that demonstrates the discriminating abilities of our portable LAMP-based system and amplification strategy presented here. Future studies must explore the extension of this diagnostic platform to the analysis of cell line co-cultures and actual biopsies from patients.

The main advantages of this LAMP-based approach are its simplicity, ease of setup and use, and speed. In contrast to other detection strategies, our system requires little expertise regarding the execution of the assays and the manipulation and interpretation of the results. For instance, although qPCR methods can be equally effective in discriminating different tissue types (as also shown here), running qPCR experiments implies an operator with more training and expertise. In addition, while qPCR amplification methods require the use of a thermocycler, the isothermal character of LAMP allows the use of a much simpler device to properly run the amplification reactions. As illustrated in this study, a very simple Arduino-based device can enable both the isothermal incubation of samples and the online measurement of the progression of the amplification reaction. The evaluation of m_P3_ values is also straightforward and demands only simple programming skills.

## Figures and Tables

**Table 1:** Sequences of the Loop-mediated Isothermal Amplification (LAMP) primers used for the detection of segments of genetic material coding for the expression of the selected genes.

Primer Set	Description	Sequence
*GAPDH*	*F3*	CTGAGAACGGGAAGCTTGTC
	*B3*	ATGGGGGCATCAGCAGAG
	*FIP*	GTACTCAGCGCCAGCATCGC-CATCACCATCTTCCAGGAGC
	*BIP*	TGGAGTCCACTGGCGTCTTCA-TGATGACCCTTTTGGCTCC
	*Loop F*	CCACTTGATTTTGGAGGGATCTC
	*Loop B*	TGGAGAAGGCTGGGGCTCAT
*ESR 1*	*F3*	CCAGAGAGATGATGGGGAGG
	*B3*	CACTGAAGGGTCTGGTAGGA
	*FIP*	TTGATCATGAGCGGGCTTGGC-GGTGAAGTGGGGTCTGCT
	*BIP*	AGAAGAACAGCCTGGCCTTGTC-GTATCGGGGGCTCAGCAT
	*Loop F*	TGGCAGCTCTCATGTCTCC
	*Loop B*	TGACGGCCGACCAGATG
*Ki67*	*F3*	GGCTGGCTTGAAAGAGCT
	*B3*	TTTGCCTGCTGATGGTGTT
	*FIP*	TGTGTCCACTGGGTCTGGTTGT-CACTGACAAGCCCACGAC
	*BIP*	GCTCCAAGCCACAGTCCAAGA-CGTTTCCTGAGTGCGAAGA
	*Loop F*	AGGCTATTTTGGTAGTTTTCTCGTG
	*Loop B*	GTCTCAGGAAAGTGGACGTAGA
*Her2*	*F3*	CAGCGGTGTGAGAAGTGC
	*B3*	GCGGGGCAGTGTTGGA
	*FIP*	TGGTAACTGCCCTCACCTCTCG-CTGTGCCCGAGTGTGCTAT
	*BIP*	GGAGTTTGCTGGCTGCAAGAAG-TGGGTCCCCATCAAAGCT
	*Loop F*	TGCTCCATGCCCAGACC
	*Loop B*	CTTTGGGAGCCTGGCATTT
*PGR*	*F3*	GCCTGAAGTTTCGGCCATAC
	*B3*	CAGTCCGCTGTCCTTTTCTG
	*FIP*	TCGTCGGAGGGGTCCTGTCC-CTATCTCCCTGGACGGGC
	*BIP*	GACGCAGGACCAGCAGTCGC-TGCTGCCTCCAGCACC
	*Loop F*	AGGGCCGAGGGAAGAGTA
	*Loop B*	GACGTGGAGGGCGCATA

**Table 2:** Tukey Pairwise Comparison of the sets of m_P3_ values determined in spheroids derived from four different cell lines. Groups with different letters are significantly statistically different from each other (Confidence level of 95% (α = 5.0%)).

	Gene			
Cell Line	*ESR 1*	*Her2*	*Ki67*	*PGR*
MCF7	A	B	B	A
MDA-MB-231	B	A	A	B
Du145	B, C	A, B	A	C
BJ	A	B	C	B

## Data Availability

The original contributions presented in this study are included in the article/Supplementary Materials. Further inquiries can be directed to the corresponding authors.

## Supplementary Materials

The following supporting information can be downloaded at: https://www.mdpi.com/article/10.3390/bios16080453/s1, Figure S1: The qLAMP Arduino-based system; Figure S2: Mechanistic basis of the electrical detection of LAMP amplification and rationale for the early-time m_P3_ readout; Table S1: PCR primers; Table S2: Variance explained by the principal components and PCA loadings for the qLAMP and RT-qPCR gene-expression datasets.
