# Supplementary material for: Quantitative Loop-Mediated Isothermal Amplification (qLAMP) for the Rapid Discrimination of Normal and Cancerous Tissue Models: An Arduino-Based Portable Cancer Detection System Assisted by a pH Microelectrode
Source: Biosensors (Basel). 2026 Aug 20;16(8):453. doi: 10.3390/bios16080453 (PMC13511430; doi:10.3390/bios16080453)
Supplement: Supplementary file 1 [file biosensors-16-00453-s001.zip › biosensors-4435593-supplementary.pdf]

Quantitative Loop-Mediated Isothermal Amplification (qLAMP) for the Rapid Discrimination of Normal and Cancerous Tissue Models: An Arduino-Based Portable Cancer Detection System Assisted by a pH Microelectrode

Supplementary information

Sergio Bravo-González [1,†], Luisa María Reyes-Cortés [1,†], Kristen Aideé Pérez-Alvarez [1], Grissel Trujillo-de Santiago [1,2,3,*] and Mario Moisés Álvarez [1,2,3,*]

[1] Centro de Biotecnología-FEMSA, Escuela de Ingeniería y Ciencias, Tecnológico de Monterrey, Ave. Eugenio Garza Sada 2501, Monterrey, NL 64700, Mexico

[2] Departamento de Mecatrónica, Escuela de Ingeniería y Ciencias, Tecnológico de Monterrey, Ave. Eugenio Garza Sada 2501, Monterrey 64700, NL, Mexico

[3] Advanced Biofabrication, Expedition-FEMSA, Tecnológico de Monterrey, Ave. Eugenio Garza Sada 2501, Monterrey 64700, NL, Mexico

* Correspondence: grissel@tec.mx (G.T.-d.S.); mario.alvarez@tec.mx (M.M.Á.)

† These authors contributed equally to this work.

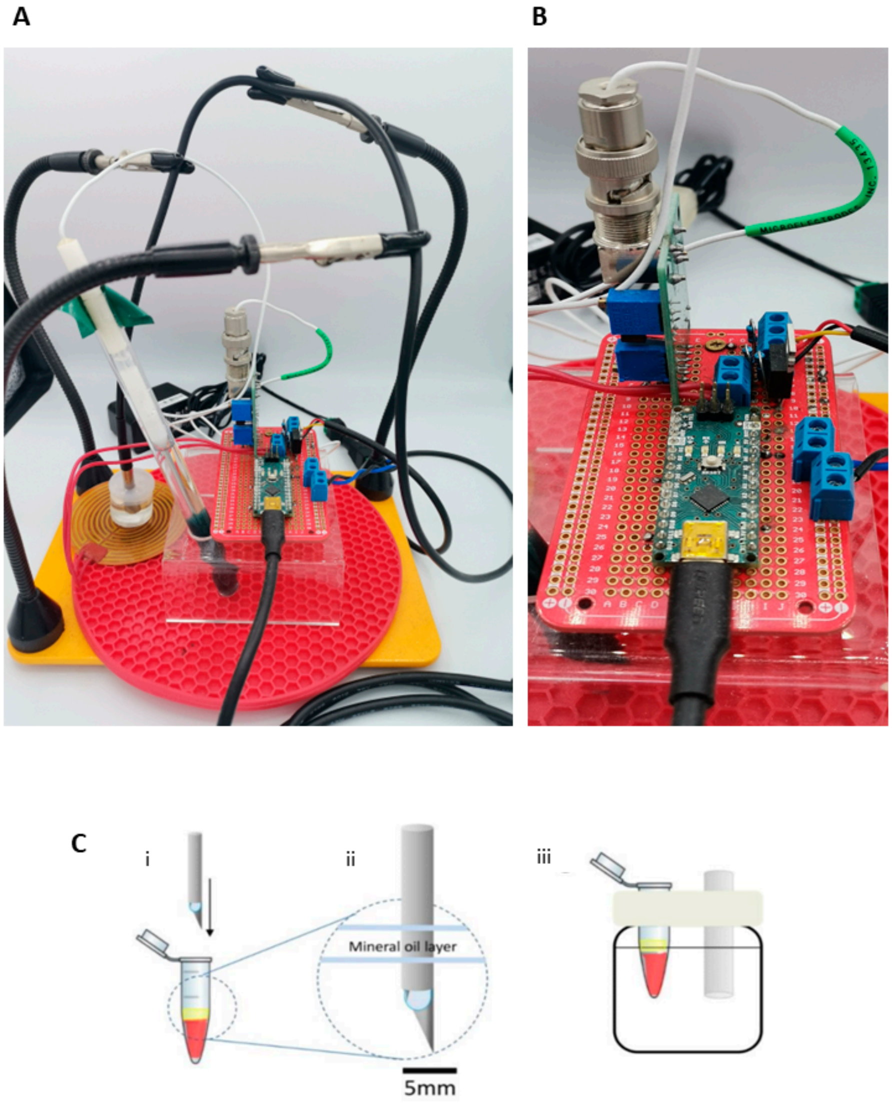

Figure S1. The qLAMP Arduino-based system. (**A**) Image of the actual system. (**B**) Close-up of the Arduino microcontroller. (**C**) Schematic representation of the oil bath and the reaction tube where the amplification occurs. (i) A micro pH electrode is inserted into the loop-mediated isothermal amplification (LAMP) colorimetric reaction mix, and (ii) a thin layer of oil is added to avoid evaporation during incubation (iii) in an oil bath.

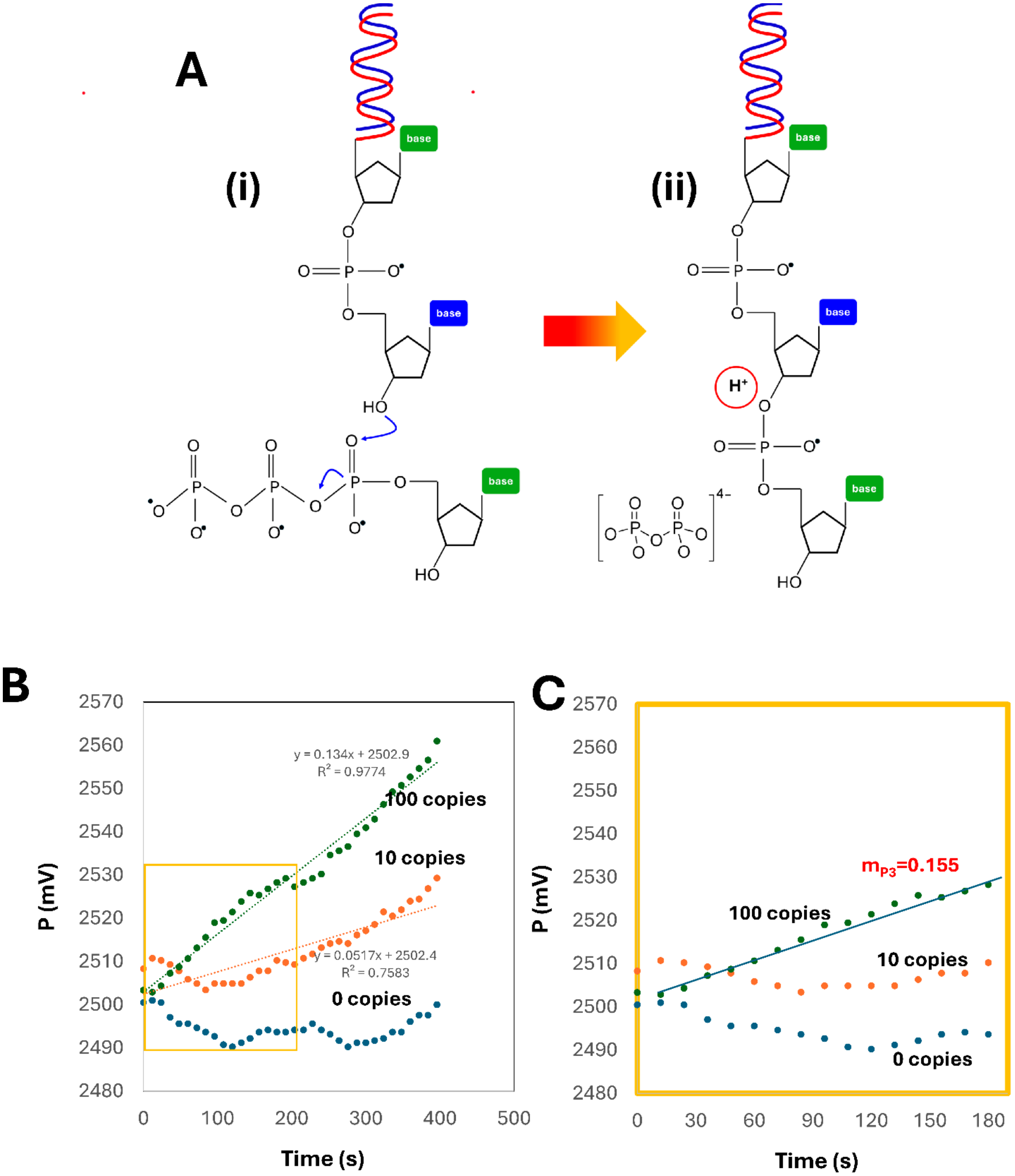

Figure S2. Mechanistic basis of the electrical detection of LAMP amplification and rationale for the early-time m_{P3} readout. (A) Schematic representation of the mechanism linking nucleic acid amplification to the electrical signal measured by the pH microelectrode. During LAMP amplification, DNA polymerase incorporates deoxynucleoside triphosphates (dNTPs) into the

growing DNA strand, with each nucleotide incorporation resulting in the formation of a phosphodiester bond and the release of pyrophosphate and a proton (H^+) into the reaction medium. Under the weakly buffered reaction conditions used in this study, progressive proton accumulation during amplification produces a decrease in pH, which is detected by the pH microelectrode as a change in electrical potential (ΔP). (**B**) Representative electrical-potential trajectories obtained during LAMP amplification using synthetic SARS-CoV-2 RNA templates containing 0, 10, or 100 copies of the target sequence. Productive amplification at 100 copies resulted in an early and sustained increase in electrical potential, whereas the 10-copy and no-template conditions exhibited substantially weaker responses. Dotted lines illustrate linear fits to the 10- and 100-copy trajectories. (**C**) Magnification of the first 180 s of the trajectories shown in panel B, corresponding to the time interval used to calculate m_{IP3}. Productive amplification at 100 copies exhibited an early approximately linear increase in electrical potential, with $m_{P3} = 0.155$ mV s^{-1}. Focusing the analysis on this early interval enables the electrical response associated with prompt target-dependent amplification to be captured while reducing the contribution of signal changes occurring at later reaction times.

Table S1. PCR primers

Primer Set	Description	Sequence
**GAPDH**	F3	CTGAGAACGGGAAGCTTGTC
	B3	ATGGGGGCATCAGCAGAG
**ESR 1**	F3	CCAGAGAGATGATGGGGAGG
	B3	CACTGAAGGGTCTGGTAGGA
**Ki67**	F3	GGCTGGCTTGAAAGAGCT
	B3	TTTGCCTGCTGATGGTGTT
**Her2**	F3	CAGCGGTGTGAGAAGTGC
	B3	GCGGGGCAGTGTTGGA
**PGR**	F3	GCCTGAAGTTTCGGCCATAC
	B3	CAGTCCGCTGTCCTTTTCTG

Table S2. Variance explained by the principal components and PCA loadings for the qLAMP and RT-qPCR gene-expression datasets.

PCA variance explained - qLAMP

Principal component	Variance explained (%)
PC1	77.03
PC2	20.65
PC3	1.83
PC4	0.49

PCA loadings – qLAMP

Biomarker	PC1	PC2
ESR1	0.660	0.187
HER2	0.609	0.369
Ki67	−0.437	0.840
PGR	0.056	0.352

PCA variance explained - qPCR

Principal component	Variance explained (%)
PC1	68.68
PC2	25.22
PC3	5.99
PC4	0.11

PCA loadings- qPCR

Biomarker	PC1	PC2
ESR1	0.346	0.648
Ki67	0.593	−0.359
HER2	0.603	−0.389
PGR	0.406	0.548
